# Betting on Your Feelings: The Interplay between Emotion and Cognition in Gambling Affective Task

**DOI:** 10.3390/jcm13102990

**Published:** 2024-05-19

**Authors:** Emanuela Mari, Clarissa Cricenti, Maddalena Boccia, Micaela Maria Zucchelli, Raffaella Nori, Laura Piccardi, Anna Maria Giannini, Alessandro Quaglieri

**Affiliations:** 1 Department of Psychology, Sapienza University of Rome, Via dei Marsi 78, 00185 Rome, Italy; clarissa.cricenti@uniroma1.it (C.C.); maddalena.boccia@uniroma1.it (M.B.); laura.piccardi@uniroma1.it (L.P.); annamaria.giannini@uniroma1.it (A.M.G.); alessandro.quaglieri@uniroma1.it (A.Q.); 2 Cognitive and Motor Rehabilitation and Neuroimaging Unit, IRCCS Santa Lucia, 00179 Rome, Italy; 3 Department of Psychology, University of Bologna, 40127 Bologna, Italy; micaela.zucchelli3@unibo.it (M.M.Z.); raffaella.nori@unibo.it (R.N.); 4 San Raffaele Cassino Hospital, 03043 Cassino, Italy; 5 Faculty of Social and Communication Sciences, Universitas Mercatorum, Piazza Mattei 10, 00186 Rome, Italy

**Keywords:** addiction, gambler, decision-making, pathological behavior, emotional states, affective states

## Abstract

**Background**: Gambling Disorder (GD) is a bio-psycho-social disorder resulting from the interaction of clinical, cognitive, and affective factors. Impulsivity is a crucial factor in addiction studies, as it is closely linked to cognitive distortions in GD by encompassing impulsive choices, motor responses, decision-making, and cognitive biases. Also, emotions, mood, temperament, and affective state are crucial in developing and maintaining GD. Gambling can be used as a maladaptive coping strategy to avoid or escape problems and distress. **Methods:** The aim of the present study is to explore differences in personality traits and emotion regulation of people suffering from GD, substance-dependent gamblers (SDGs), and healthy controls (HCs). Additionally, the study proposes a new experimental task: the “Gambling Affective Task” (GAT) to investigate the influence of affective priming on risk-taking behaviors. **Results**: Our findings indicate that participants placed lower bets following positive priming. Additionally, SDGs wagered significantly higher amounts than HCs, regardless of priming type. In general, participants exhibited longer response times after positive priming trials, compared to negative and neutral priming trials. These findings suggest that experiencing positive emotions can act as a protective factor by delaying and lengthening gambling behaviors. By comparing gamblers with and without substance comorbidity, we can gain insight into the exclusive factors of GD and improve our understanding of this disorder. **Conclusions**: By elucidating the impact of emotional states on risk-taking, the research also provides new insights into the prevention and treatment of GD.

## 1. Introduction

Gambling Disorder (GD) appears to result from a combination of factors interacting at different levels [1] and can be considered a bio-psycho-social disorder [2,3]. Firstly, according to the literature review, individuals with previous psychopathological disorders, such as substance abuse, mood, anxiety, and personality disorders, are more likely to suffer from GD [1,4,5]. Furthermore, individuals with GD tend to exhibit specific personality traits, such as neuroticism or emotional instability [6]. These traits, along with impulsivity and emotional dysregulation, may increase the risk of engaging in gambling activities [7]. When gambling is used as a maladaptive coping strategy (e.g., to avoid or escape problems and distress), affective factors seem to underlie the manifestation of GD [8]. In this context, the anticipation of excitement from gambling can increase the likelihood of addiction, leading to more frequent gambling and resulting in more opportunities for loss and increased symptoms of GD [1]. In addition to clinical disorders and affective factors, cognitive alterations also significantly contribute to the development and maintenance of GD. Specifically, impaired judgment and decision-making processes can result in cognitive distortions, such as the illusion of control or the gambler’s fallacy (i.e., erroneous beliefs about the probability of winning and the contribution of one’s abilities to winning in outcomes that depend on chance). These distortions can have significant impacts [9,10]. They are also associated with the persistence of GD by prompting continued behavior despite frequent losses [11] and a diminished perception of risk, resulting in a more optimistic view of outcomes [12]. In this paper, we aim to explore the impact of impulsivity, emotional states, and affect on Gambling Disorder (GD). We will begin by reviewing existing evidence in this area. Subsequently, we will present a new experimental study that seeks to determine whether affective states can directly influence the decision-making processes related to risk-taking in people with GD, Substance-Dependent Gamblers (SDGs), and healthy individuals.

### 1.1. Impulsivity

Impulsivity represents a key factor in GD and is “the inability to resist an urge or temptation even if it is harmful to oneself or others”, referring to impulsive, risky, and inappropriate behavior that leads to negative consequences [13]. Impulsivity has always been a central variable in the study of addictions [14,15,16]. From a cognitive perspective, impulsivity is characterized by components such as impulsive choices, or “discounting” (i.e., the preference for smaller but immediate rewards rather than larger but delayed rewards), a central aspect of decision-making; impulsive motor responses (i.e., the inability to suppress inappropriate motor responses); impulsive decision-making (i.e., risky choices in ambiguous situations); reflexive impulsivity (i.e., the tendency to make premature responses under conditions of high uncertainty); and impulsive cognitive bias (i.e., the failure to suppress inappropriate attentional biases) [17,18]. Impulsivity in GD is also linked to cognitive distortions (e.g., the illusion of control), associated with an impulsive decision-making style that leads individuals to accept erroneous beliefs [19]. In some respects, GD is associated with a lack of cognitive control, which includes various processes such as response inhibition, conflict monitoring, decision-making, and cognitive flexibility. Impulsivity is one of the aspects of this lack of control observed in gamblers [20,21]. Specifically, deficits in response inhibition seem to be involved in the development and perpetuation of GD [22], as well as in increased GD severity [23]. Indeed, impulsivity may be an expression of decreased cognitive control [24]. A recent meta-analysis [25] aimed to detect the association between GD and different dimensions of impulsivity. The results revealed a strong association between GD and motor inhibition [23,26,27], attention inhibition [28], delay discounting [29], and impulsive decision-making [26,30].

### 1.2. Emotional and Affective States

Different terms have been used to describe different affective states. These terms include emotions, mood, temperament, and affective style. They are often referred to as “emotional competence”, encompassing the skills of emotional regulation. Both affective states and emotional competence play a key role in the development and persistence of addictive disorders. The term “affect” encompasses a wide range of emotional experiences, including values, preferences, and attitudes (i.e., affective dispositions), as well as specific emotions and moods [31]. In particular, “emotion” refers to a composite, multicomponent construct indicating an intense affective state or reaction with acute onset and transient character. The complex nature of emotions is demonstrated by their cognitive evaluations of stimuli, neural/psychophysiological responses, and psychological, experiential, and subjective components [32,33]. Since the early 1980s [34,35], studies aimed at identifying the structure of affective states have emphasized the presence of two main factors, generally referred to as positive and negative affect. Although these may suggest the presence of two opposite states, they can actually be considered as two distinct and unrelated dimensions. Specifically, positive affect or activation reflects how much a person feels enthusiastic, active, and alert. Therefore, a high level of positive affect represents a state of energy, concentration, and engagement, while low levels of positive affect represent a state of sadness and lethargy. Conversely, negative affect or activation reflects a general dimension of subjective distress that may include different aversive emotional states, such as anger, contempt, disgust, guilt, fear, and nervousness. Low levels of negative affect are, therefore, characteristic of states of calm and serenity [36].

### 1.3. The Effect of Affective States on Decision-Making Processes

Emotions and affects have different functions in an individual’s life. They help people cope with the basic tasks of life, communicate feelings to others, facilitate relationships, and influence actions. For example, anticipating the emotional experience that will serve as an incentive to enact an instrumental action is a motivational function of emotions and affects [37].

In terms of decision-making, emotions’ influence on motivation is crucial. Immediate vs. anticipated emotions distinction is important [38]. Anticipated emotions refer to an individual’s expectations of the emotions experienced due to choice. When making decisions, people tend to select emotions that will maximize positive feelings or minimize negative ones. Immediate emotions are the individual’s affective state at the time of decision-making. Studies, such as those conducted by Hudson and colleagues [39], have examined the impact of specific emotional states on the attentional processes related to risky behaviors, such as engaging in gambling activities. Slovic and Peters [40] suggested that emotions play a role in decision-making, as they can influence how we evaluate alternatives and lead us to choose things we like and avoid those we dislike. However, decision-making processes can also be influenced by incidental affective states (i.e., those not related to the characteristics of the object).

The study of affect and decision-making has gained particular relevance due to its connection to avoidance or risk-taking behavior. The direction in which positive and negative emotions and affective states influence decision-making and risk-taking is unclear. According to the Mood Maintenance Hypothesis (MMH; [41]), individuals with positive affective states or moods avoid taking risks to maintain the positive affective state, while those with negative affective states or moods tend to engage in more risk-taking behavior to increase their positive affective state. Conversely, according to the Affect Infusion Model (AIM; [42]), affect influences risk-taking through an effect congruent with mood: a positive affective state primes individuals to be more vulnerable to access thoughts about positive aspects of risky situations, perceiving the outcome of risky choices as more favorable and possible, leading to more risk-seeking behavior, while a negative affective state, by increasing accuracy in information processing and perceiving more threat, leads to risk avoidance.

Studies in this area have reported inconsistent results [43,44,45,46,47,48]. However, despite these inconsistencies, the literature agrees on the crucial role that affect plays in decision-making processes and the subsequent enactment of risky behavior. According to decision-making literature, behavior is shaped by the interplay of two cognitive processes: System I, which is automatic, intuitive, fast, experiential, and affect-based, and System II, which is controlled, analytical, slow, deliberative, and logical [49]. According to the dual-process theory, addiction behavior involves a competition between these two automatic and controlled systems. The former system leads to substance use, while the latter system refrains from it [50]. This theory also applies to gambling behaviour, where the impulse to bet and the ability to refrain from it are in conflict. Both addiction and gambling populations show deficits in controlled processes such as executive control and inhibition functions [22,51]. As a result, gambling cues, such as betting shops, are likely to capture attention and elicit approach behaviors. The inability to consider the positive long-term consequences of refraining from betting, until behavior becomes mainly controlled by automatic processes, can make it difficult to recover from pathological behavior. The individual can overcome this by favoring the prevalence of controlled processes over the automatic impulse to bet.

In contrast, a more dynamic approach called the value-based decision-making model (VBDM; [52]) has been proposed and applied to the addiction framework. This model suggests that pathological behavior is determined by comparing its benefits/costs to all other possible behaviors. Evidence for each specific option accumulates, integrating information about the overall utility of that option, including positive and negative consequences, cost, effort, etc., competing over time until one reaches a threshold, and the action is implemented [53]. When faced with the choice between engaging in the target behavior, such as betting or not, an enhanced evidence accumulation and lower response thresholds for betting occur compared to increased response thresholds and blunted evidence accumulation for the alternative behavioral option. Recovery from pathological behavior, in this case, occurs when evidence accumulation for the target action is suppressed while it is amplified for the alternative option and the response threshold increases.

### 1.4. The Present Study

Most of the available literature has analyzed the psychopathology within Gambling Disorder as a whole. However, few studies have focused on identifying specific alterations in different types of pathological gamblers; indeed, much of the literature presents gamblers with at least one other comorbid psychiatric disorder. To our knowledge, few studies have compared these variables between “pure” gamblers and substance-using gamblers. Although “pure” gamblers constitute only a small part of the pathological context, they offer the opportunity to identify and analyze exclusive or common characteristics between the two types of gamblers. Blaszczynski and Nower’s “Pathway model” [2] has underscored the importance of identifying the motivation that drives individuals to gamble, emphasizing the emotional component, and identifying specific characteristics for each type of gambler, which underlie the development and maintenance of gambling behavior. In this model, in “emotionally vulnerable” gamblers, it is the emotion that could more significantly explain the pathogenesis of addiction, representing a group of “high-functioning” gamblers not affected by the neurotoxic effect of substances.

Therefore, the purpose of the present study is to extend the findings of a previous study of ours [54], in which it was found that the “pure” GD group differed from the SDG group primarily in terms of mood disorder traits such as depression, anxiety, and hostility. To this end, different emotional and affective dimensions have been included, along with the creation of an affective task, with the aim of shedding light on both the emotional aspects and their impact on risk-taking decision-making processes.

Specifically, we have developed a novel experimental task known as the Gambling Affective Task (GAT), in order to delve into the influence of positive and negative affective priming on risk-taking behaviors. Our aim was to shed some light on this matter, as the existing literature lacks a unanimous agreement on whether positive or negative affective states should be regarded as a risk or protective factors in the development and maintenance of GD.

## 2. Materials and Methods

### 2.1. Participants and Procedure

GD and Substance-Dependent Gamblers (SDGs) were recruited from two distinct recovery communities specialized in treating individuals with GD and SDG. Both communities offer 24/7 resident monitoring treatment programs. Healthy controls (HCs) were recruited using convenience sampling and carefully matched to the experimental groups based on demographic characteristics. They were selected from the official website of the University of Rome “Sapienza”. None of the participants in the control group met the criteria outlined in the Diagnostic and Statistical Manual of Mental Disorders (5th ed.; DSM-5; [55]) for current GD or any form of drug addiction. 

In the initial analysis, the diagnosis of GD or SDG was made by assessing specific National Health System facilities in the region and subsequently confirmed by psychiatrists working within the recovery communities based on DSM-5 criteria. Recruitment of GD and SDG individuals involved specific exclusion criteria, including psychotic spectrum disorders, progressive neurodegenerative disorders, neurological diseases, inability to provide informed consent, or complete required assessment procedures. Both experimental groups were in treatment, abstinent from GD and substances, and received psychoeducational and psychotherapeutic interventions for gambling addiction. Additionally, participants in the experimental groups (GD and SDG) were required to report a score equal to or higher than 5 on the South Oaks Gambling Screen (SOGS; [56]). To be eligible to participate in the SDG group, individuals had to score 3 or higher on the Drug Abuse Screening Test-10 (DAST-10; [57]) or 8 or higher on the Alcohol Use Disorder Identification Test (AUDIT; Piccinelli [58]).

A priori power analysis was conducted in G*Power (Version 3.1.9.4) to determine the adequate sample size to obtain a statistical power of 0.95 to observe a medium effect size (f ≥ 0.25). This power analysis determined that a sample of *N* = 66 would be required to obtain a power of 0.95 under these parameters. The total sample consisted of 64 participants, including 24 individuals with GD, 20 individuals with SDG, and 20 healthy controls (HCs). All participants were men aged between 19 and 59 years (M = 35.89 years; SD = 11.78 years) (see Table 1).

The participants did not show any significant age differences (F_(2,61)_ = 0.282; *p* = 0.755) but were not equally distributed about educational level (χ^2^_(8)_ = 27.234; *p* < 0.01), as the HC group more frequently included participants with a higher educational level than the other two groups. 

**Figure 1:**
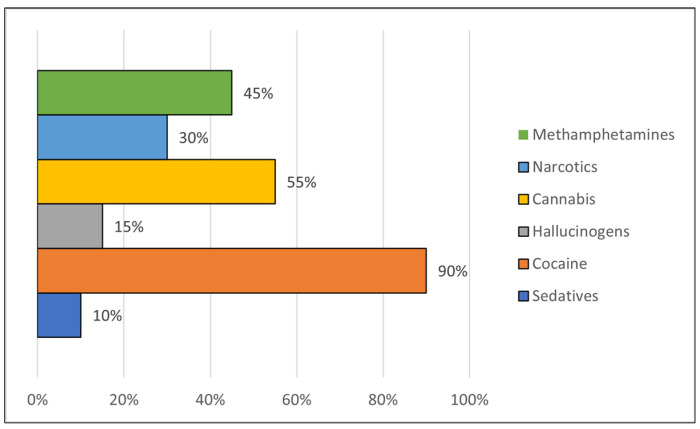
Substance use habits in comorbid gamblers.

Concerning the screening scales (i.e., South Oaks Gambling Screen—SOGS; Alcohol Use Disorder Identification Test—AUDIT), GD individuals obtain higher mean difference in the SOGS than both SDG (M = 2.38, SE = 0.944, *p* < 0.001) and HC (M = 12.98, SE = 0.944, *p* < 0.001); also, SDG individuals obtain higher mean difference than GD (M = 11.53, SE = 2.25, *p* < 0.001) and HC (M = 11.00, SE = 2.35, *p* < 0.001) in the AUDIT. Participants in the SDG group also had an average score of 6.85 on the DAST-10 test, confirming the presence of other substance addictions in comorbidity with GD (see Figure 1 and Table 2).

During the first meeting, the study was introduced briefly. After ensuring that participants were willing to take part in the study, the aims, goals, and confidentiality of the procedure were explained in detail. Participation in the study was voluntary and there was no compensation for taking part. All participants underwent assessments to evaluate addiction characteristics, addictive behaviors, impulsivity, and cognitive functions (i.e., decision-making). 

Initially, anamnestic, clinical, and subclinical information was collected through individual interviews conducted by a psychologist and psychotherapist. These interviews were conducted in agreement with the research team to ensure uniformity in the information gathered from participants. Residents provided information about their personal history and the motivations that led them to develop Gambling Disorder. Participants with neurological and/or psychiatric disorders were excluded from the interviews. Participants alternated in administering tasks and questionnaires in a pseudo-random manner and completed the entire procedure within the day, considering the already scheduled needs and commitments of the communities. After the procedure, feedback on the study’s purposes and aims was provided. All study procedures were conducted in accordance with the Declaration of Helsinki. The Institutional Review Board of the University of Rome “Sapienza” (protocol number 221/2020) approved the procedures and accompanying consent forms.

### 2.2. Questionnaires

#### 2.2.1. South Oaks Gambling Screen (SOGS, Italian Version, [56])

It is a self-reported 20-item questionnaire based on DSM-III criteria for GD. Individuals should indicate their adherence or non-adherence to the described behavior; answers are then summed, with a score of 5 or higher indicating potential GD. In this study, the Italian version of the SOGS was used and reported to have a Cronbach’s alpha of 0.94.

#### 2.2.2. Drug Abuse Screening Test (DAST, [57])

This is a screening questionnaire developed to identify drug-related problems. The DAST-10 version is a short version of the original 28-item questionnaire. It is based on 10 questions concerning information on involvement with drugs, excluding alcohol and tobacco. Participants should indicate their adherence or non-adherence to the described behavior. All answers are summed with higher scores, which indicate severe levels of drug-related problems. It showed good reliability with Cronbach’s Alpha of 0.86. 

#### 2.2.3. Alcohol Use Disorder Identification Test (AUDIT, [58])

It is a fast-screening test developed by the World Health Organization (WHO) to identify at-risk drinkers and alcohol-related problems. In Italy, it was validated by Piccinelli and colleagues [58]. It comprises 10 items on a 4-point Likert scale ranging from 0 (never) to 4 (daily or almost daily). A cut-off score of 5 was associated with a sensitivity of 0.84, a specificity of 0.90, and a predictive value of 0.60 [58]. It showed good reliability with Cronbach’s Alpha of 0.84

#### 2.2.4. Barratt Impulsiveness Scale (BIS-11, Italian Version, [59])

This is a self-reported 30-item questionnaire developed to assess the personality and behavioral dimension of impulsiveness, describing both impulsive and non-impulsive behaviors. The questionnaire measures three second-order factors of impulsivity: attentional impulsiveness (in turn defined by attention and cognitive instability first-order components); motor impulsiveness (in turn defined by motor and perseverance components); and non-planning impulsiveness (in turn defined by self-control and cognitive complexity components). All items are measured on a 4-point Likert scale ranging from 1 (rarely/never) to 4 (almost always/always). The items are summed, and the higher the total score, the higher the impulsiveness level. The Italian version developed by Fossati, and colleagues [59] showed a good fit index with the original factor structure, with a Cronbach’s alpha of 0.79.

#### 2.2.5. Toronto Alexithymia Scale (TAS-20, Italian Version, [60])

It is one of the most common measures of alexithymia, a multifaceted personality construct that represents a deficit in the cognitive processing of emotion. Participants are encouraged to evaluate twenty items on a 5-point Likert scale ranging from 1 (strongly disagree) to 5 (strongly agree). The TAS-20 results in a total score and three subscale scores (i.e., Difficulty Identifying Feelings, Difficulty Describing Feelings, and Externally Oriented Thinking). Higher scores on the TAS-20 indicate greater difficulties in the cognitive processing of emotion. In this study, we used the Italian version of the TAS-20 [60], which showed good reliability with a Cronbach’s alpha of 0.75. 

#### 2.2.6. Difficulties in Emotion Regulation Scale (DERS-36, [61])

This is a self-reported questionnaire that assesses six dimensions of emotion regulation (i) Clarity reflecting the extent to which individuals understand the emotions they are experiencing, (ii) Non-acceptance reflecting a tendency to have negative secondary emotional responses to one’s negative emotions, (iii) Lack of Emotional Awareness reflecting difficulties to attend to and acknowledge emotions, (iv) Impulse reflecting difficulties remaining in control of one’s behavior when experiencing negative emotions, (v) Goals reflecting difficulties concentrating and accomplishing tasks when experiencing negative emotions, (vi) Strategies reflecting the difficulty to regulate emotions effectively once an individual is upset. Items are rated on a scale of 1 (almost never) to 5 (almost always). Higher scores indicate severe difficulty in emotion regulation. The Cronbach’s alpha coefficients for dimensions exceeded 0.88 and ranged from 0.84 to 0.92. 

#### 2.2.7. Emotion Regulation Questionnaire (ERQ, Italian Version, [62])

This is a self-reported 10-item questionnaire rated on a 7-point Likert scale from 1 (strongly disagree) to 7 (strongly agree). It consists of two dimensions: Cognitive Reappraisal and Expressive Suppression. These two dimensions are considered two strategies for regulating emotion and are reported as important in emotion management. In this study, we used the Italian version developed by Balzarotti and colleagues [62], which showed good reliability with an alpha of 0.84 and 0.72 for the Reappraisal and Suppression scale, respectively.

#### 2.2.8. Positive and Negative Affective Schedule (PANAS, Italian Version, [63])

This is a 20-item (i.e., 10-positive and 10-negative adjectives) that measures the two main dimensions of affective experience. In this study, we adopted the state time version by instructing participants to report the extent to which they feel “at this moment” as described by each of the 20 adjectives, using a 5-point Likert scale ranging from 1 (very slightly or not at all) to 5 (extremely). The Italian version showed good reliability for positive (PA) with an alpha of 0.83 and negative (NA) with an alpha of 0.87.

### 2.3. Behavioral Tasks

#### 2.3.1. Iowa Gambling Task (IGT, [64]) 

It is a neurocognitive task used to measure decision-making processes under ambiguous conditions. The task consists of a card game in which participants must select a card from one of four available decks for 100 trials. Each deck can be associated with a gain or loss of money. The loss of money is higher in the A or B decks and lower in the C or D decks. Therefore, decks A and B are “disadvantageous”, with the highest long-term risk and loss, while decks C and D are “advantageous” and return a small long-term gain or loss. Participants start with €2000 of virtual money and are instructed to maximize their profit. We calculated the mean Net score for each 25-trial block (i.e., the number of choices from good decks C and D minus the number of choices from bad decks A and B). This result is calculated to quantify the change and to monitor the participant’s progressive learning curve during the task, according to the learning curve provided by the authors of the task [64].

#### 2.3.2. Game of Dice Task (GDT, [65])

It is a task used to measure decision-making and risk-taking. The GDT is similar to the IGT but differs in its explicit risk condition of gaining or losing. Participants start with €1000 of virtual money and are instructed to maximize their profit. The probability of winning or losing is associated with each of the 18 trials during the GDT. Participants are instructed to bet on a single die face, or combinations of two, three, and four die faces simultaneously. Betting on only one side of the dice potentially produces a gain or loss of €1000 (i.e., the probability of winning is 1:6), betting on the combination on two sides of the dice simultaneously produces a gain or loss of €500 (i.e., the probability is 2:6), betting on the combination on three sides of the dice simultaneously produces a gain or loss of €200 (i.e., the probability is 3:6), and betting on the combination on four sides of the dice produces a gain or loss of €100 (i.e., the probability is 4:6). Choosing to bet on the single or two-sided combination of the dice (i.e., associated with less than 50% probability of winning and high gains/high losses) can be considered as risky choices while choosing to bet on the three- or four-sided combination of the dice is considered relatively safe choices. Although GDT is similar to IGT, the main difference between the two is that the probability of winning/losing is presented explicitly during GDT, whereas the probabilities are implicit in IGT until the participant deduces them through trial and error during the task. According to this criteria we computed different variables, including the total number of wins (N_wins), total number of losses (N_losses), number of risky choices after a win (RaW), number of risky choices after a loss (RaL), number of safe choices after a win (SaW), number of safe choices after a loss (SaL), a total number of risky choices (N_R), and a total number of safe choices (N_S).

#### 2.3.3. Gambling Affective Task (GAT; Figure 2)

This is a new experimental task designed for the present study. It was implemented using OpenSesame software (version 4.0.5) and was aimed at investigating how affective priming can influence gambling choices. The GAT was implemented in Rome (Italy) by the Laboratory of Applied Experimental Psychology, Department of Psychology, “Sapienza” University of Rome. The task consists of 90 trials, each of which is preceded by a picture (with a duration of 250 ms) extracted from the International Affective Picture System (IAPS). The IAPS was developed by the National Institute of Mental Health-Center for Emotion and Attention at the University of Florida [66]. The pictures were selected as emotional activators following an analysis of variance (ANOVA) between the mean values for each of the three identified conditions (i.e., 30 pictures with positive affective valence, 30 pictures with neutral affective valence, and 30 pictures with negative affective valence), to select pictures with valence and arousal as homogeneous as possible between the different stimuli, excluding those that were extremely activating and those that were not activating at all. Each trial includes the possibility of betting against the “dealer”, with a choice of the bet amount between €0, €10, €25, €35, or €50 (i.e., where zero is equivalent to moving on to the next trial). We analyzed the response time latency taken by participants to place their bet and the amount of virtual money wagered for each condition (i.e., after a negative, positive, or neutral priming); for these two variables, we computed the safe and risky conditions in a similar way to the GDT, by considering a bet of 10 or 25 as a relatively safe choice and a bet of 35 or 50 as a risky choice. 

**Figure 2:**
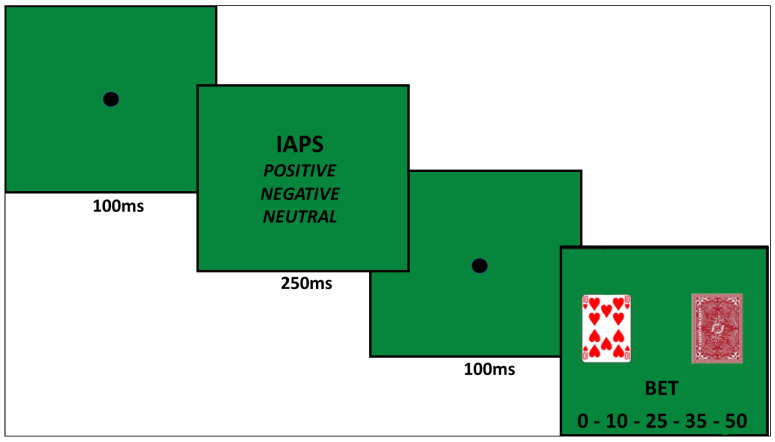
Gambling Affective Task.

### 2.4. Statistical Analysis

Statistical analyses were performed using SPSS software (version 26). First, the reliability of the instruments was tested using Cronbach’s Alpha; the results showed high reliability with an alpha ranging from 0.72 to 0.94. Participants were divided into three groups and compared through clinical and cognitive measures. For each variable, the normality assumption was tested by analyzing the skewness and kurtosis indices and conducting the Shapiro test. For the clinical variables, where the assumption of normality was met, differences between groups were examined using analysis of variance (MANCOVA) followed by post hoc comparisons with Bonferroni correction. The educational level reported statistically significant results between groups and, therefore, was included as a continuous covariate (i.e., the total number of school years, with a mean value of 11.81) to control for possible differences in the sample that may play a role in specific clinical variables (e.g., impulsivity). Between-group differences in cognitive tasks (i.e., IGT and GAT) were examined using a general linear model (GLM) with repeated measures, in which the between-subjects factor was the specific group assignment (i.e., HC, GD, and SDG) and the within-subjects factor was the scores obtained on the different trials. The dependent variables were the scores obtained by participants in the IGT task and those obtained in the GAT task. Concerning the GDT task, differences between groups were analyzed using one-way analysis of variance (ANOVA), followed by post hoc comparisons with a Bonferroni correction. In addition, bivariate correlations (i.e., Pearson’s r coefficient) were conducted. Statistical significance was defined as *p* < 0.05.

## 3. Results

### 3.1. Impulsivity 

The data analysis revealed statistically significant differences between groups concerning mean scores in the second-order sub-dimensions of BIS-11: motor impulsivity (F_(2,63)_ = 8.98; *p* < 0.001, η2 = 0.230) and non-planning impulsivity (F_(2,63)_ = 10.55; *p* < 0.001, η2 = 0.260), and not a statistically significant result for attentional impulsivity (F_(2,63)_ = 2.44; *p* > 0.05). The educational level covariate did not show statistically significant results (*p* = 0.900; 0.711; 0.875) for attentional, motor, and non-planning impulsivity, respectively. Post-hoc comparisons showed that the mean scores of the HC group were significantly lower than those of the GD and SDG in both motor impulsivity and non-planning impulsivity. Pairwise differences between groups for attentional impulsivity scores were not significant after the post-hoc comparisons (Table 3).

### 3.2. Emotional and Affective States

Concerning the emotional and affective states, we found a significant difference between groups in the negative sub-dimension of PANAS (F_(2,63)_ = 3.534; *p* < 0.05, η2 = 0.105), but the educational level covariate did not show a statistically significant result (*p* = 0.675; 0.589) for positive and negative affect sub-dimension, respectively. However, the adjustment for multiple comparisons with Bonferroni correction results revealed no difference between groups. All comparisons are reported in Table 4.

### 3.3. Cognitive Aspects Underlying Decision-Making and Risk-Taking

To investigate group differences in both the IGT and GAT, the general linear model (GLM) with repeated measures was used with the group as the between-subjects factor (i.e., HC, GD, and SDG) and NET-scores (i.e., average scores obtained in the 4 different NETs), or the RT and BET-scores for GAT as a within-subjects factor. With respect to the GDT, a one-way ANOVA analysis was used for each outcome (i.e., N_wins; N_losses; RaW; RaL; SaW; SaL; N_R; N_S).

### 3.4. Iowa Gambling Task

**Figure 3:**
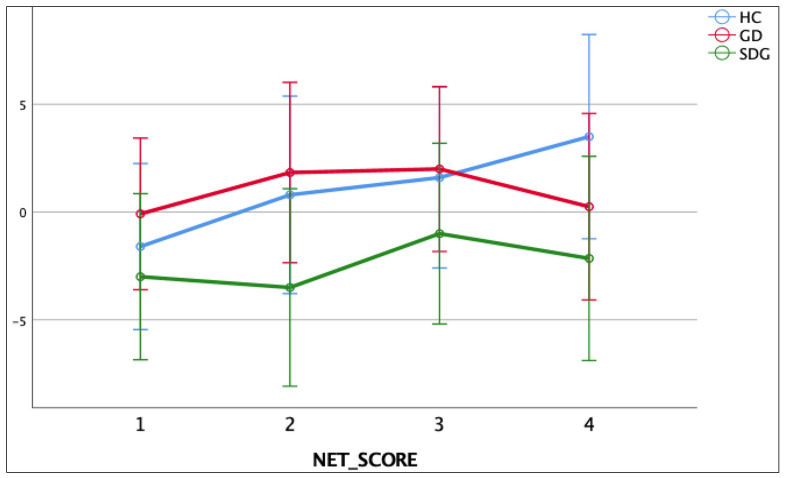
Group comparisons on Net scores of Iowa Gambling Test.

Specifically, in the IGT, a 3 × 4 factorial design (groups × NET) was used. The results revealed no statistically significant effects between subjects (group; F_(2,61)_ = 1.591; *p* = 0.212; η2 = 0.050), nor within subjects (NET; F_(2,61)_ = 1.278; *p* = 0.284; η2 = 0.021) and with respect to interaction (F_(2,61)_ = 0.601; *p* = 0.693; η2 = 0.019). The results obtained are summarised in Figure 3.

### 3.5. Game of Dice Task

With regard to GDT, one-way ANOVA analysis was performed and showed that there were statistically significant differences in the variables number of risky choices after a loss (i.e., RaL) (F_(2,61)_ = 3.462, *p* < 0.5), number of safe choices after a loss (i.e., SaL) (F_(2,61)_ = 7.534, *p* < 0.01), the total number of risky choices (i.e., N_R) (F_(2,61)_ = 3.179, *p* < 0.05), and the total number of safe choices (i.e., N_S) (F_(2,61)_ = 3.540, *p* < 0.05). Post-hoc comparisons revealed statistically significant differences between groups about the variable “RaL” where the SDG group scored higher on average than the HC group. Concerning the variable “SaL”, the SDG group scored on average lower than the HC group. For the “N_S”, it emerges that the SDG group obtained lower average scores relative to the HC group (Table 5).

### 3.6. Gambling Affective Task

With regard to the GAT task, a 3 × 3 factorial design (groups × valence) was applied concerning both amounts bet (i.e., BET) and response times (i.e., RT). Concerning the amounts participants bet, the results showed a statistically significant main effect of the valence (F_(2,122)_ = 3.138; *p* < 0.05; η2 = 0.050), while the interaction effect between valence and groups was not significant. Pairwise comparisons revealed a statistically significant mean difference, with neutral valence reporting higher values (M = 28.11, ES = 11.17, *p* < 0.05) than positive ones (Table 6).

**Figure 4:**
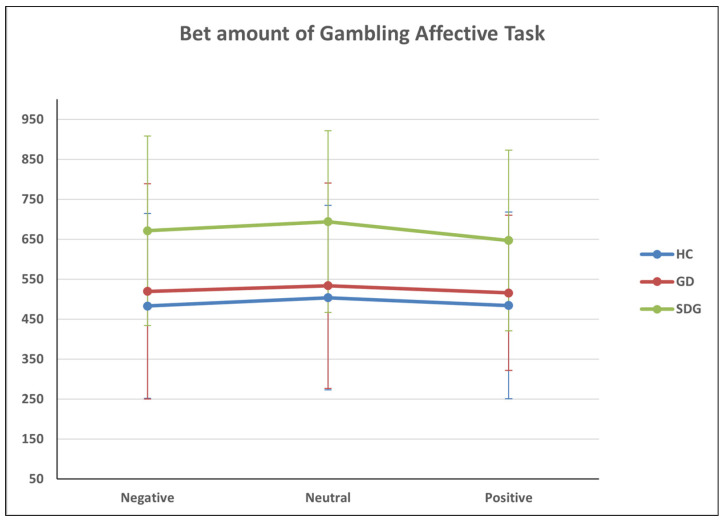
The GAT_BET variable.

The between-subjects analysis also revealed a statistically significant difference between groups (F_(2,60)_ = 3.544, *p* < 0.05; η2 = 0.106). Post-hoc comparisons revealed a statistically significant difference between SDG and HC, with the SDG group betting a significantly higher amount (M = 180.17, SE = 72.55, *p* < 0.05) than the HC group (Figure 4).

**Figure 5:**
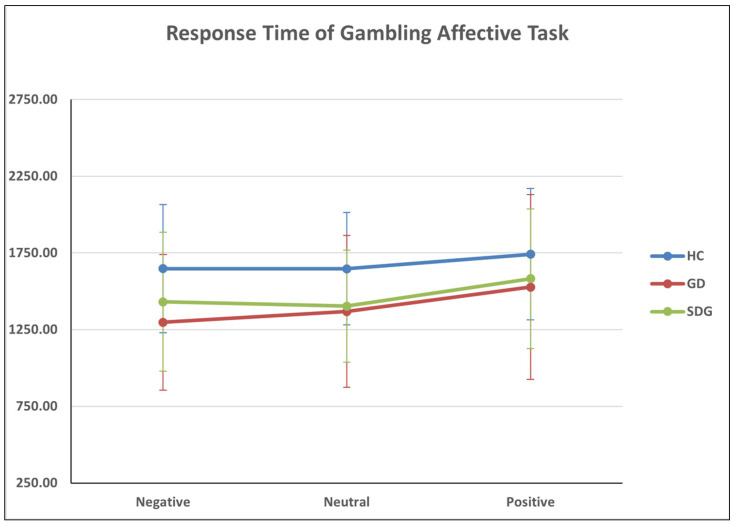
The GAT_RT variable of the Gambling Affective Task.

With respect to the response time, results showed a statistically significant main effect of the valence (F_(2,102,75)_ = 8.974, *p* < 0.001; η2 = 0.130). While both the interaction effect of valence and group (*p* = 0.629) and between-subjects effect (*p* = 0.084) were not significant. Through pairwise comparisons, a statically significant difference was found between positive and negative valence (M = 158.382, SE = 46.840, *p* < 0.01) and between positive and neutral valence (M = 144.614, SE = 43.847, *p* < 0.01) (Table 7, Figure 5).

#### Amount of Risky and Safety Bets

For participant’s risk-taking propensity, we separately analyzed the amount of bet (i.e., the sum of the risky bet) for high-risk or less conservative bets (i.e., only trials where participants placed a 35 or 50 coin bet) and safety or more conservative bets (i.e., only trials where participants placed 10 or 25 coin bets). 

The results showed a statistically significant main effect of valence (F_(2,114)_ = 3.932, *p* < 0.05; η2 = 0.065), no interaction effect between valence and groups (*p* = 0.888), and there was a statistically significant result of between-subjects effect (F_(1,57)_ = 4.248, *p* < 0.05, η2 = 0.130). Pairwise comparisons showed that the mean difference was lower in trials following positive affective priming (M = −39.13, SE = 14.51, *p* < 0.05) than after neutral affective priming. Concerning the post-hoc tests, the SDG group placed more risk bets (M = 203.19, SE = 73.35, *p* < 0.05) than HC. 

Concerning the response time related to risky bets, there was a statistically significant main effect of valence (F_(2,85,81)_ = 13.638, *p* < 0.05; η2 = 0.193), no interaction effect between valence and groups (*p* = 0.258), and there was a statistically significant result of between-subjects effect (F_(1,57)_ = 4.863, *p* < 0.05, η2 = 0.146). Pairwise comparisons showed a lower mean difference when comparing Neutral than Negative (M = −182.546, SE = 58.13, *p* < 0.01) and Positive (M = −431.313, SE = 83.933, *p* < 0.001). The HC group scored higher than both GD (M = 354.487, SE = 132.75, *p* < 0.05) and SDG group (M = 371.080, SE = 134.320, *p* < 0.05). 

For safe bets (i.e., trials with 10 or 25 coin bets), there were no statistically significant results for the main effect of valence (*p* = 0.108), interaction effect (*p* = 0.406), and between-subjects effect (*p* = 0.617). Also, for the response time, there were no statistically significant results for the main effect (*p* = 0.097), interaction effect (*p* = 0.264), and between-subjects effect (*p* = 0.100).

### 3.7. Convergent Validity of Gambling Affective Task

Firstly, we conducted a correlation analysis to compare variables between GAT, GDT, and IGT. Each variable of GDT was subjected to Pearson’s correlation with each complementary variable of GAT. The GAT_BET overall was positively correlated with a total number of risky choices of GDT (r = 359, *p* < 0.01), and negatively correlated with a total number of safe choices of GDT (r = −0.427, *p* < 0.01). Also, the Risky_BET of GAT (i.e., only trials with 35 or 50 coin bet) was positively correlated with the total number of risky choices of GDT (r = 0.368, *p* < 0.01), with the number of risky choices taken by participants after a win (r = 0.397, *p* < 0.01) and after a loss (r = 0.311, *p* < 0.05), and negatively correlated with safe choices taken after a win (r = −0.447, *p* < 0.01), and with the total number of safe choices of GDT (r = −0.426, *p* < 0.01). Lastly, Safe_BET of GAT (i.e., only trials with 10 or 25 coin bets) was positively correlated with the NET_1 score that corresponds at advantageous choices made by participants during trials comprised between 1–25 of IGT. Concerning the Risky BET of GAT were also found positive correlations with attentional impulsivity (r = 0.272; *p* < 0.05), DERS strategies (r = 0.435; *p* < 0.01), DERS clarity (r = 0.357; *p* < 0.01), TAS difficulties in identifying feelings (r = 0.410, *p* < 0.01), TAS externally-oriented thinking (r = 0.421, *p* < 0.01), and negatively correlated with ERQ reappraisal (r = −0.429, *p* < 0.01), and PANAS positive (r = −0.414, *p* < 0.01).

## 4. Discussion

The aim of this study was to investigate several factors associated with the development and maintenance of GD, including impulsivity, mood disorders, and substance use comorbidity. Despite that these factors are already known in the literature as underlying GD, it is uncommon to compare gamblers with and without substance comorbidity [67]. This is because the use of other substances can be confusing in attributing factors that play a role in gambling behaviour. Previous studies have highlighted neurobiological alterations that appear to be independent of substance intake [68] and have shown the presence of exclusive factors (e.g., Gambling Disorder severity, mood disorder) in a sample of “pure” gamblers in-treatment [54]. The lack of adaptive emotion regulation strategies appears to modulate the relationship between personality traits and gambling severity, in which gambling may represent a way to suppress negative affective states and escape personal needs (i.e., gambling as a coping strategy) [69]. In line with the research’s overall goal, a preliminary finding has been obtained regarding the severity of gambling, measured through the South Oaks Gambling Screen-SOGS. The results show that “pure” gamblers had higher average scores than substance-dependent gamblers (SDG). This outcome could be attributed to the fact that, in our study, all SDG participants were gamblers as well. However, they likely considered gambling as a secondary addiction that frequently developed as a result of substance dependency [54].

Concerning clinical variables, both experimental groups (i.e., “pure” gamblers and gamblers with comorbidities of other addictions) were found to differ from the control group in terms of two dimensions of impulsivity [25]. These results seem to be in line with the crucial role played by impulsivity in the development of addictions [14,15,70,71]. Individuals with Gambling Disorder often exhibit impulsive decision-making and cognitive distortions, such as illusion of control, which lead them to accept false beliefs [19]. Cognitive distortions, such as overconfidence in one’s ability to predict gains and losses or alterations in retrospective interpretations of what caused them (i.e., bad luck), represent further relevant components in manifesting GD [72]. These distortions are strongly linked to deficits in emotional regulation and lead the individual to consider the consequences of gambling abnormally. As evidenced by our results, the negative affect could modulate the relationship between specific personality traits (e.g., impulsivity) and gambling severity [73]. Individuals with Gambling Disorder may find gambling a way to suppress intolerable emotional states (e.g., shame or guilt), but also to escape personal needs that they are unable to express in intimate relationships [69]. Some studies [74,75,76] have also shown that adaptive emotional regulation strategies are overrepresented in the population of gamblers and positively correlated with the severity of gambling-related cognitive distortions. For example, using “positive refocusing” (i.e., turning attention away from the emotion) would appear to lead the individual to overestimate their self-efficacy, increasing symptom severity and cognitive distortions [74]. In particular, the propensity to positively re-evaluate negative situations (e.g., loss) to reduce their emotional impact may be dysfunctional because it reduces contact with negative emotions that might signal the need to change behavioral strategy (e.g., stop gambling after a loss), rather than re-evaluating the situation as positive (e.g., loss predicts future winnings). 

Concerning the assessment of neurocognitive variables, we used two well-known gambling tasks (i.e., Iowa Gambling task and Game of Dice). These tasks shared some common risk-taking mechanisms but had different risk conditions (i.e., the explicit and ambiguous risk conditions) that involve executive functions differently: the ambiguous condition (i.e., Iowa gambling task) seems to involve executive functions in a less demanding way. In fact, tasks in which explicit rules are provided seem to recruit more cognitive processes (e.g., working memory and executive control). Poor performance on the Iowa Gambling Task has been widely reported in the literature as a measure of impaired decision-making in different neurological and psychiatric conditions [77,78,79,80,81]. Although our results are not statistically significant, the study showed worse performance (e.g., during the last NET score), especially in comorbid gamblers, supporting a possible impairment of decision-making processes related to risk-reward, impulsivity, and poor cognitive flexibility [82,83]. Concerning the performance on the Game of Dice Task, the “pure” gamblers group performed similarly to the control group; however, the comorbid gamblers group showed poor performance. This finding suggests that comorbid gamblers may have a greater aversion to delay discounting by showing a preference for risky choices (i.e., with a higher payoff) with a low probability of winning. This leads to a worsening of the overall score based on both the total number of safe and risky choices after a loss (i.e., a long-term negative balance). Previous studies using GDT suggested a high correlation with specific executive functions (e.g., cognitive flexibility), which might play a key role in risky decision-making, suggesting an underlying dysfunctional frontal activity (e.g., the orbitofrontal cortex [67,84]). This finding seems to be more dysfunctional in comorbid gamblers due to the permanent neurotoxic effects caused by substances [85,86]. In this regard, for the decision-making models discussed in the introduction section that have been applied to addiction behavior and advanced for gambling behavior as well, the results of the study appear to be more supportive of a dynamical VBDM model in which the decision to bet is based on the progressive integration of different information, rather than on a static competition of two automatic and controlled boosts. However, to be confirmed this suggestion deserves further studies. 

Interesting data emerges from the new task implemented in our study (i.e., Gambling Affective Task); this task was specifically created to investigate the influence of affective priming (i.e., positive, negative, and neutral, respectively) on gambling choices. Our results showed a lower bet amount after a positive priming than in trials preceded by a neutral priming. Also, when considering group results, comorbid gamblers wagered higher than the healthy control group, regardless of the type of priming. Regarding the response times, it was found that, following positive priming, participants had longer response times than negative and neutral priming. Taken together, these findings seem to align with previous studies [87] that investigated reaction times about positive and negative emotional activators, suggesting that the activation of positive emotions could be considered a protective factor as it would lengthen and delay the game conduct. In fact, according to the Mood Maintenance Hypothesis (MMH [43]), people who experience positive affect are less likely to participate (or participate to a lesser extent) in risk-taking behavior than people in a neutral affective state [88]. This occurs as people do not want to “risk” their positive affective state in addition to their bet and are more risk averse as a means of sustaining their positive mood state. Similarly, according to the “Affect regulation” hypothesis [89], people who experience negative mood make more risky decisions to improve their emotional state; in other words, individuals might be more likely to behave in a riskier way because their attention is more directed on the unlikely positive outcomes rather than the more likely negative outcomes [90,91]. From a mood repair perspective, it can be expected that people in a negative mood will choose risky options to achieve a positive outcome that could improve their state. This result seems to disregard changes in processing strategies. For example, theorists such as Frijda [92], have argued that emotion has a primary motivational function, which helps to regulate actions by signaling the presence or absence of threatening states, thus negative mood states (signaling the presence of a problem) should promote analytical processing through the use of rational and elaborative strategies directed at avoiding problems and consequently reducing risky choices.

Another aim of our study was to verify the convergent validity between the GAT and GDT tasks using correlation analysis. In line with what was expected, many convergent correlations were found between complementary variables of both tasks and with different clinical variables. For example, the number of “Risky” choices of GDT showed positive correlations with the impulsivity scale, emotional (dys)regulation, and alexithymia scale, and negative correlations with the reappraisal dimension of the ERQ scale. Concerning clinical variables, results showed positive correlations between dimensions of impulsivity and different dimensions of emotional state. These correlations supported the complex interaction occurring in Gambling Disorder in which sensation seeking and excitement, risk acceptance, and the emotion of gambling become dispositional factors that act as rewards. Gamblers with higher severity of the disorder are more likely to gamble impulsively and experience great excitement, which produces a reinforcing effect of the gambling compulsion. Gamblers cannot wait for the reward; their gratification cannot be delayed but must be immediate and quick, as stated by different delay-discounting paradigms [93,94,95]. This leads the individual to develop a sense of restlessness and irritability because the next pay-out takes a long time, and physical and mental agitation may also occur. A gambler who cannot win and at the same time cannot stop gambling establishes a vicious circle that leads the individual into a state of anxiety and depression. When disorders such as depression or anxiety are already present before the development of gambling addiction, they can trigger or precipitate the addiction [96].

The primary aim of the study was to extend, to the emotional dimension, the presence of unique features in “pure” gambling addiction that could differentiate these gamblers from those with substance comorbidities. The importance of detecting differences in “pure” gamblers lies in the possibility of understanding which characteristics are proper and resulting from behavioral addiction exclusively without the confounding effect of neurotoxic substances. Although several neuroimaging studies support a clear overlap of many alterations in executive and cognitive functions in gambling and substance use disorders [67,97,98,99], “pure” gamblers might represent a class of high-functioning gamblers with the ability to maintain the integrity of some superior functions. This specific group of gamblers seems to reflect the characteristics of the “emotionally vulnerable” gambler provided by the “Pathway model” of Blaszczynski and Nower [2]. These gamblers are less likely to exhibit comorbidity with substances, gamble more often to modulate negative affective states, and use gambling as a coping strategy [8].

In conclusion, this research offers new insights and raises questions regarding “pure” gamblers but shows some limitations. First, it is important to consider a selection bias associated with convenience sampling, suggesting the need to be careful when interpreting and generalizing the data. Since it appears to be very challenging to find gamblers who manifest and maintain gambling as a primary addiction, the limited sampling may have limited the representativeness of the type and level of comorbidity in the general population, leading to possible erroneous conclusions on the relationship between gambling, comorbidity, and personological variables. Furthermore, it is important to note that all participants were tested at different times concerning their permanence in the rehabilitation communities. However, all were tested within one year of entering the rehabilitation community. Despite interviews and anamnestic investigations being conducted to rule out the participants’ possible neurological or psychiatric disorders, the present study did not investigate variables related to intelligence quotient (IQ), probably affecting the interpretation of results during behavioral tasks. Also, results provided by the new decision-making task GAT can only be considered partially, as there was no assessment of attentional vigilance (e.g., Simple Vigilance Test-SVT) that could provide another concurrent validity for the task.

## 5. Conclusions

The findings highlighted specific traits in GD, which can be observed independently of comorbidity with other substances, suggesting the need to investigate further the comparison between “pure” and comorbid gamblers with substance addiction. Considering the already discussed limitations of the research, a future aim could be to replicate the study with a larger and more representative sample. It would also be important to carry out a longitudinal study during the different phases of community treatment by monitoring the trend of both clinical and cognitive variables over time. However, consistent with the data, this study supports the crucial role played by emotions and affective states in both the development and maintenance of Gambling Disorder. A specific protocol was created ad hoc for this study (i.e., Gambling Affective Task GAT) to investigate both the effect of specific affective state induction (i.e., positive, negative, or neutral) on gambling behavior and the implementation of a scenario as ecological as possible. The GAT was found to be a valid and reliable computerized task for the brief assessment of decision-making under non-explicit risk in a clinical sample of individuals with gambling disorders. With more facets of decision-making being captured than in the IGT and GDT (i.e., the effect of affective priming on decision-making), relatively short task duration (about 20 min), and pleasing design, it is a recommended task for research on decision-making in population-based and clinical samples, specifically samples with GD. Future studies could be conducted on gambling preferences (i.e., strategic and non-strategic) within the different groups of gamblers to investigate whether any correlations exist between personological aspects and the type of gambling preferred. 

The results of this study indicate that gambling is a complex phenomenon, and it is necessary to investigate it more closely. It is also important to develop specific therapies for different types of gamblers. To do so, it is crucial to understand their primary motivations for gambling and tailor the intervention accordingly. The research materials and testimonies collected can help raise awareness and provide training for young people and adults. Prevention efforts should focus on both cognitive and emotional aspects to bring about a cultural change. Unlike other substance addictions, gambling addiction is not easy to detect without the necessary tools to understand its dynamics.

## Figures and Tables

**Table 1:** Distribution of the sample by age and educational qualification.

		GD Means (SD)	SDG Means (SD)	HC Means (SD)	F	*p*
Age		37.33 (11.18)	35.10 (11.88)	34.95 (12.80)	0.282	0.755
					χ^2^	*p*
Educational level	Primary school	0 (0%)	1 (5%)	0 (0%)	27.234	0.001
	Secondary school 1°	9 (37.5%)	11 (55%)	0 (0%)		
	Secondary school 2°	15 (62.5%)	7 (35%)	13 (65%)		
	Bachelor’s degree	0 (0%)	1 (5%)	3 (15%)		
	Master’s degree	0 (0%)	0 (0%)	4 (20%)		

**Table 2:** Addiction Screening Measures Scores.

	GD Means (SD)	SDG Means (SD)	HC Means (SD)	F	*p*
SOGS	13.33 (3.54)	10.95 (3.97)	0.35 (0.59)	15.866	<0.001
AUDIT	2.17 (2.70)	13.70 (12.91)	2.70 (1.46)	103.364	<0.001
DAST-10	-	6.85 (1.87)	-	-	-

Note. SOGS = South Oaks Gambling Screening; AUDIT = Alcohol Use Disorder Identification Test; DAST-10 = Drug Abuse Screening Test; GD = Gambling Disorder; SDG = Comorbid Gambling Disorder; HC = Healthy control.

**Table 3:** Group comparisons for impulsivity scores.

	Groups	M	SD	F	*p*	η2
BIS-11(II) Attentional Impulsivity	HC	12.55	3.27	2.435	0.096	0.075
	GD	14.96	3.37			
	SDG	14.55	3.07			
BIS-11(II) Motor Impulsivity	HC	17.90	3.45	8.979	<0.001	0.230
	GD	24.42	6.06			
	SDG	24.65	4.67			
BIS-11(II) Non Planning Impulsivity	HC	22.25	4.49	10.554	<0.001	0.260
	GD	29.54	5.36			
	SDG	29.15	4.30			

**Table 4:** Group comparisons of emotional and affective states.

	Groups	M	SD	F	*p*	η2
DERS Non acceptance	HC	12.00	4.90	1.679	0.195	0.053
	GD	13.75	5.25			
	SDG	12.40	4.44			
DERS Goals	HC	12.60	486	0.210	0.811	0.007
	GD	11.50	4.10			
	SDG	11.95	3.25			
DERS Strategies	HC	14.50	5.46	1.251	0.294	0.040
	GD	17.25	7.52			
	SDG	18.50	6.50			
DERS Impulse	HC	9.80	3.87	1.473	0.238	0.047
	GD	12.00	5.32			
	SDG	13.45	5.47			
DERS Clarity	HC	9.25	3.95	2.019	0.142	0.063
	GD	11.58	5.68			
	SDG	12.70	4.04			
DERS Awareness	HC	12.45	3.75	1.839	0.168	0.058
	GD	15.88	5.23			
	SDG	15.60	3.36			
PANAS Positive	HC	34.95	6.45	0.189	0.828	0.006
	GD	35.79	7.37			
	SDG	35.75	5.93			
PANAS Negative	HC	20.35	5.24	3.534	0.035	0.105
	GD	27.21	9.61			
	SDG	22.40	7.88			
ERQ Reappraisal	HC	30.50	7.21	0.150	0.861	0.005
	GD	27.67	8.77			
	SDG	27.45	9.53			
ERQ Suppression	HC	16.05	5.46	0.636	0.533	0.021
	GD	13.04	6.69			
	SDG	13.30	4.99			
TAS Difficulty identifies feeling	HC	12.90	5.17	1.077	0.347	0.035
	GD	16.96	7.92			
	SDG	17.95	6.21			
TAS Difficulty Describing Feelings	HC	13.55	5.49	1.158	0.321	0.037
	GD	15.29	6.20			
	SDG	13.60	4.63			
TAS External-oriented thinking	HC	16.00	4.41	1.879	0.162	0.059
	GD	18.00	5.78			
	SDG	19.80	4.77			

**Table 5:** Group comparisons for average scores of Game of Dice Task.

	Group	M	SD	F	*p*	η2
N_wins	HC	10.30	1.867	0.841	0.436	0.027
	GD	9.46	3.092			
	SDG	9.20	3.238			
N_losses	HC	7.70	1.867	0.841	0.436	0.027
	GD	8.54	3.092			
	SDG	8.80	3.238			
RaW	HC	0.60	1.046	1.433	0.246	0.045
	GD	1.25	1.595			
	SDG	1.35	1.843			
RaL	HC	1.45	1.356	3.462	0.038	0.102
	GD	3.63	4.470			
	SDG	4.40	4.210			
SaW	HC	9.10	2.269	1.187	0.312	0.039
	GD	8.50	3.700			
	SDG	7.40	4.321			
SaL	HC	5.85	1.348	7.534	0.001	0.203
	GD	4.73	1.486			
	SDG	3.85	2.007			
N_R	HC	2.40	2.210	3.179	0.049	0.094
	GD	5.38	5.948			
	SDG	6.20	5.827			
N_S	HC	15.60	2.210	3.540	0.035	0.107
	GD	13.77	4.710			
	SDG	11.80	5.827			

Note. N_wins = Number of wins; N_losses = Number of losses; RaW = Number of risky choices after a win; RaL = Number of risky choices after a loss; SaW = Number of safe choices after a win; SaL = Number of safe choices after a loss; N_R = Total number of risky choices; N_S = Total number of safe choices.

**Table 6:** Descriptive statistics of GAT_BET.

	Group	Mean	SD
BET_NEGATIVE	HC	483.25	8.26
	GD	519.56	8.63
	SDG	671.25	7.86
	Total	556.19	256.51
BET_NEUTRAL	HC	504.00	227.26
	GD	533.70	257.13
	SDG	694.00	230.99
	Total	575.16	249.93
BET_POSITIVE	HC	484.50	226.31
	GD	515.87	194.47
	SDG	647.00	233.77
	Total	547.54	225.18

**Table 7:** Descriptive statistics of GAT_RT variable.

	Group	Mean	SD
RT_NEGATIVE	HC	1647.81	418.28
	GD	1297.78	441.76
	SDG	1431.36	452.71
	Total	1451.31	454.97
RT_NEUTRAL	HC	1646.8	365.75
	GD	1368.72	494.53
	SDG	1402.71	364.92
	Total	1467.80	428.89
RT_POSITIVE	HC	1741.59	428.59
	GD	1527.82	602.53
	SDG	1582.70	455.32
	Total	1613.10	506.92

Note. The averages reported in the table are the original values.

## Data Availability

Data will be made available on reasonable request.
